# Physiologically Based Pharmacokinetic Modelling with Dynamic PET Data to Study the* In Vivo* Effects of Transporter Inhibition on Hepatobiliary Clearance in Mice

**DOI:** 10.1155/2018/5849047

**Published:** 2018-06-03

**Authors:** Marco F. Taddio, Linjing Mu, Claudia Keller, Roger Schibli, Stefanie D. Krämer

**Affiliations:** ^1^Radiopharmaceutical Science and Biopharmacy, Institute of Pharmaceutical Sciences, Department of Chemistry and Applied Biosciences, ETH Zurich, Zurich, Switzerland; ^2^Department of Nuclear Medicine, University Hospital Zurich, Switzerland

## Abstract

Physiologically based pharmacokinetic modelling (PBPK) is a powerful tool to predict* in vivo* pharmacokinetics based on physiological parameters and data from* in vivo* studies and* in vitro* assays.* In vivo* PBPK modelling in laboratory animals by noninvasive imaging could help to improve the* in vivo-in vivo* translation towards human pharmacokinetics modelling. We evaluated the feasibility of PBPK modelling with PET data from mice. We used data from two of our PET tracers under development, [^11^C]AM7 and [^11^C]MT107. PET images suggested hepatobiliary excretion which was reduced after cyclosporine administration. We fitted the time-activity curves of blood, liver, gallbladder/intestine, kidney, and peripheral tissue to a compartment model and compared the resulting pharmacokinetic parameters under control conditions ([^11^C]AM7 *n* = 2; [^11^C]MT107, *n* = 4) and after administration of cyclosporine ([^11^C]MT107, *n* = 4). The modelling revealed a significant reduction in [^11^C]MT107 hepatobiliary clearance from 35.2 ± 10.9 to 17.1 ± 5.6 *μ*l/min after cyclosporine administration. The excretion profile of [^11^C]MT107 was shifted from predominantly hepatobiliary (CL_H_/CL_R_ = 3.8 ± 3.0) to equal hepatobiliary and renal clearance (CL_H_/CL_R_ = 0.9 ± 0.2). Our results show the potential of PBPK modelling for characterizing the* in vivo* effects of transporter inhibition on whole-body and organ-specific pharmacokinetics.

## 1. Introduction

Clinical drug-drug interactions result in many cases from an inhibition of drug-transporting proteins in the liver or kidney [1–4]. Hepatocytes express a variety of drug-relevant transporter proteins. Transporters such as OATP1B1 (encoded by* SLCO1B1*) and OATP1B3* (SLCO1B3)* can facilitate drug entry into the hepatocytes and, therefore, promote drug metabolism. Efflux transporters, such as P-glycoprotein* (ABCB1)* and BCRP (*ABCG2*), can transport their substrates, including drugs and their metabolites, from the hepatocytes into bile, which is secreted into the small intestine [2]. Inhibition of drug-transporting proteins can consequently reduce both drug metabolism and drug or metabolite excretion into bile.

In the glomeruli of the kidneys, drugs are filtrated out of the plasma into the primary urine. Lipophilic drugs are reabsorbed from the tubuli upon the concentration of the primary urine. In addition to filtration/reabsorption, transport proteins in the proximal tubuli can promote the transport of drugs from blood into urine [2]. The renal clearance (CL_R_) results from glomerular filtration, reabsorption, and tubular secretion. Transporter inhibition may thus reduce CL_R_ besides hepatobiliary clearance (CL_H_).

Whether a drug is a substrate and/or an inhibitor of a particular transporter protein can be studied* in vitro* with transporter-overexpressing cells [5, 6]. However, the* in vivo* consequences are not always easy to predict. Physiologically based pharmacokinetic modelling (PBPK) to predict* in vivo* kinetics based on* in vitro* data requires detailed information on the expression levels and activity of individual transport proteins in both the* in vitro* model and the* in vivo* organisms [7, 8]. Information is particularly limited for laboratory animals as rodent transporter-overexpressing cells and* in vitro-in vivo* weighting functions for modelling are scarcely available [9, 10].

Dynamic noninvasive imaging by positron emission tomography (PET) or single photon emission computed tomography (SPECT) allows assessing the hepatobiliary or renal clearance of a suitable tracer in humans and laboratory animals. In most cases, the data are analysed with simplified, robust models, focusing on one particular elimination process. Clinical examples are the evaluation of transporter-mediated hepatocyte uptake and efflux into bile with (15*R*)-[^11^C]TIC-Me or the conjugated bile acid tracer [^11^C]CSar [11, 12]. We explored the possibility of studying the effects of transporter inhibition on the overall pharmacokinetics of a radiolabelled molecule by PET in mice. PET allows generating well-defined tissue radioactivity concentration time curves (*C*(*t*)) from image data. In addition, blood can be sampled to measure the blood* C*(*t*) simultaneously [13, 14]. PET kinetic modelling is a standard method for the quantification of brain function, for example, glucose consumption or neuroreceptor density in preclinical and clinical imaging [13–15]. Here, we shift the focus of PET kinetic modelling from the study of an individual process or organ to* in vivo* whole-body PBPK modelling in mice.

**Figure 1:**
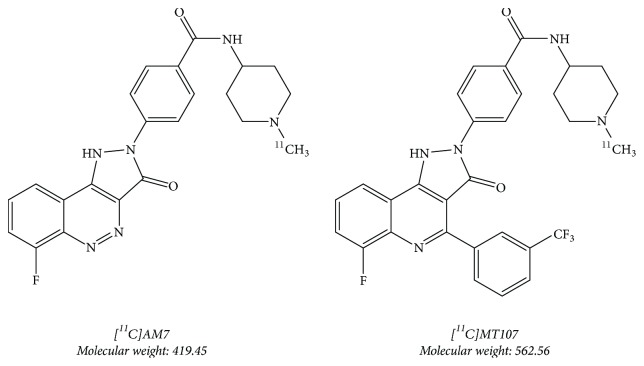
[^11^C]AM7 and [^11^C]MT107.

In this feasibility study, we repurposed mouse PET data gathered with our PET tracers under development [^11^C]AM7 and [^11^C]MT107, both targeting the human costimulatory molecule CD80 (hCD80) [16] (Taddio et al., in preparation; Figure 1). CD80 is a surface protein on activated antigen-presenting cells (APCs). Upon antigen presentation, its interaction with CD28 activates T cells while binding to CTLA-4 inactivates T cells and depletes CD80 from the cell surface of the APCs [17, 18]. By targeting CD80, we aim to image elevated immunogenic activity, for example, in cancer, atherosclerosis, or autoimmune diseases. In our previous work with [^11^C]AM7, we observed high biliary excretion of radioactivity resulting in high radioactivity spill-over from the abdomen. The tracer furthermore showed low tissue uptake, in agreement with its low lipophilicity, log⁡*D* (pH 7.4) of 0.1, and low unbound fraction in plasma (*f*_u_) 0.02 [16]. As a consequence, accumulation in hCD80-positive xenografts was negligible. The structurally modified [^11^C]MT107 (Figure 1), based on structures by Green et al. [19] and Huxley et al. [20], had a similar strong affinity in the low nanomolar range to the imaging target hCD80 as [^11^C]AM7. Its log⁡*D* (pH 7.4) was higher with 2.0 and albumin binding was similar to that of [^11^C]AM7 (Taddio et al., in preparation). PET images showed higher tissue radioactivity compared to [^11^C]AM7 but still high abdominal radioactivity accumulation. In this study, we investigated the pharmacokinetics of the two tracers by* in vivo* PBPK modelling and studied the effects of transporter inhibition by cyclosporine on the pharmacokinetics of [^11^C]MT107. We show that* in vivo* PBPK modelling in mice is possibly based on dynamic whole-body PET data.

## 2. Materials and Methods

### 2.1. Small Animal PET with [^11^C]AM7 and [^11^C]MT107

[^11^C]AM7 and [^11^C]MT107 (Figure 1) were synthesized as described previously for [^11^C]AM7 [16]. The synthesis of the precursor for [^11^C]MT107 was based on Green et al. (2003) and a patent from 2004 [19, 21] (Taddio et al., in preparation). Molar activities at the end of synthesis were between 200 and 500 GBq/*μ*mol for [^11^C]AM7 and between 20 and 80 GBq/*μ*mol for [^11^C]MT107.

Animal experiments were in accordance with the Swiss legislation on animal welfare and approved by the Veterinary Office of the Canton Zurich, Switzerland. For this study, we used PET data from 7-to-10-week-old female C.B.17 SCID or CD1 nude mice (16.9–21.2 g body weight; Charles River, Sulzberg, Germany), carrying hCD80-positive Raji xenografts according to [16, 22]. Raji cells were from DSMZ (Braunschweig, Germany). The xenograft-related results will be published elsewhere (Taddio et al., in preparation). As the radioactivity fraction in the xenografts was negligible compared to the total radioactivity dose, tracer distribution to the xenografts was not taken into account in the modelling.

For PET/CT acquisition, mice were anaesthetized with 3 to 5% isoflurane in air/oxygen (1 : 1), at a respiratory rate of ~60 per min as described elsewhere [16]. Cyclosporine (50 mg/kg), to inhibit cyclosporine-sensitive transporters, was injected into a tail vein (i.v.), 30 to 50 min before tracer injection while the respective tracer was synthesized. The injected cyclosporine solution was a dilution with water for injection (1 : 1) of Sandimmun® (Novartis Pharmaceuticals, Basel, Switzerland; 50 mg/ml cyclosporine in 26.1% ethanol/65% PEG-35 castor oil). Vehicle (13% ethanol, 2 ml/kg) was injected as a control as indicated. Immediately after radiosynthesis quality control, the tracer was injected i.v. at a dose between 3 and 14 MBq (<20 nmol/kg) in 100 to 200 *μ*L saline containing 5% ethanol. Injections lasted ~10 s. The mouse was transferred to a SuperArgus PET/CT scanner (Sedecal, Madrid, Spain, formerly Vista eXplore) with an axial field of view of 4.8 cm and a spatial resolution of 1.6–1.7 mm (full width at half maximum; [23]). Body temperature and respiratory rate were controlled as previously described [16]. The PET scan was started in List mode 60 s after tracer injection. After 60 min scan duration, computed tomography (CT) data were recorded for anatomical orientation.

Two mice were scanned with [^11^C]AM7 under control conditions (with vehicle injection) and one was scanned with [^11^C]AM7 after cyclosporine administration. Group sizes for [^11^C]MT107 were *n* = 4 for control conditions (one with vehicle and three without vehicle) and *n* = 4 after cyclosporine administration. At the end of the PET and CT scans, the mice were euthanized by decapitation, still under isoflurane anaesthesia, and two [^11^C]AM7 and one [^11^C]MT107 mice were dissected to measure tissue radioactivities in a gamma counter (1480 Wizard 3′′, Perkin Elmer).

The PET data were reconstructed into 10 or 12 time frames by 2D Fourier rebinning/ordered-subsets expectation maximization (FORE/OSEM), 2 iterations, and 16 subsets, correcting for singles and randoms but not attenuation. Images were generated with the software PMOD v3.8 (PMOD, Zurich, Switzerland). All radioactivities were decay-corrected to the time point of tracer injection.

### 2.2. Time-Activity Curves

The blood *C*(*t*) was generated with PMOD from the PET images as follows. A cropped cube of 10 × 10 × 10 mm^3^ including the image data of the heart was divided into 12 segments with differing kinetics, using the PSEG module of PMOD. *C*(*t*) of the segment covering the left heart ventricle according to the PET (first time frame)/CT images was used as an estimation of the blood *C*(*t*) (*C*_Blood_(*t*)). *C*_Blood_(*t*) was divided by (1 − hematocrit) to get *C*_Plasma_(*t*) and fitted to a biexponential infusion function as shown in

(1) CPlasmat=A0T×V1×λ1×V1/Vz−λ1λ1λz−λ1×1−e−λ1×tt≤T+Tt>T×e−λ1×t−Tt>T+λ1×V1/Vz−λzλzλ1−λz×1−e−λz×tt≤T+Tt>T×e−λz×t−Tt>T,

where *A*(0) is the radioactivity dose, *T* is the duration of the injection (10 s infusion), *V*_1_ is the volume of the central compartment (initial volume of distribution of the tracer after injection), *V*_*z*_ is the volume of distribution during the terminal phase, and *λ*_1_ and *λ*_*z*_ are the respective rate constants of the biexponential function and *t* the time [24]. The term [*t*(*t* ≤ *T*) + *T*(*t* > *T*)] equals *t* if *t* ≤ *T* but *T* if *t* > *T*. The term (*t* − *T*)(*t* > *T*) equals *t* − *T* if *t* > *T*, otherwise zero. The hematocrit was assumed 0.44 [25, 26].

Total plasma radioactivity (*A*_Plasma_(*t*)) was estimated as the product of the image-derived *C*_Blood_(*t*) and the theoretical blood volume (*v*_Blood_ 0.0585 ml per g body weight, BW [25]) multiplied with BW. Blood-related data may be biased by radioactivity spill-over and partial volume effects.

*C*(*t*) of liver, kidneys, and peripheral tissue and *A*(*t*) of gallbladder and intestines were derived from manually drawn volumes of interest according to the PET/CT images, using the VOI functions of PMOD. Regions of interest are shown for a representative scan in Supplementary Figure 1. *C*(*t*) data were transformed to *A*(*t*) by multiplication with the reported average volume of the respective organ or tissue. These were 0.065 cm^3^ per g BW for liver and 0.0164 cm^3^ per g BW for kidneys [26]. The volume of the “peripheral tissue” (*V*_Tissue_) with *C*(*t*) determined from the left shoulder was estimated during the fitting procedure. It was 0.74 ± 0.08 cm^3^ per g BW for [^11^C]MT107 control scans and 0.79 ± 0.10 cm^3^/g for [^11^C]MT107 scans after cyclosporine treatment, without significant difference (*p* = 0.47).

### 2.3. Pharmacokinetics Model and Nonlinear Least-Squares Curve Fitting

**Figure 2:**
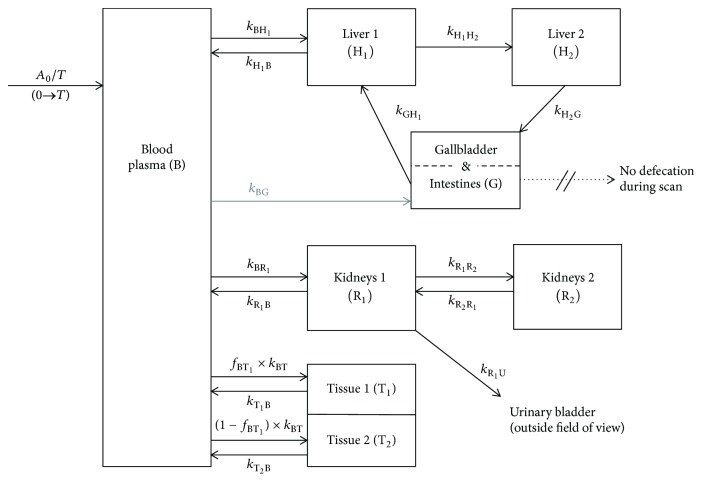
Model for the pharmacokinetic analysis. *A*(0) is the dose, and *T* is the infusion duration (10 s). Initial *A*(*t* = 0) for all compartments (indicated by black boxes) were zero. The parameters *k* are mass transfer rate constants with the unit 1/time. The indices denote the source and target compartments, respectively (e.g., *k*_BH_1__, *k* for the mass transfer from blood plasma to hepatic compartment “Liver 1”). *A*(*t*) of peripheral tissue was best fit with two sets of rate constants for reversible transfer. The sum of the two plasma-to-tissue rate constants is *k*_BT_. Several models were evaluated and the results were visually inspected. The shown model revealed reliable fits as concluded from the robust fit parameters and the visual inspection of the plotted fit functions. For [^11^C]MT107 scans, *k*_BG_ (grey arrow) was set to 0. Tissue blood fractions (*v*_Blood_ multiplied with the organ or tissue volume and *C*_Blood_(*t*)) were added to the compartments where applicable. B, blood plasma; G, gallbladder plus intestine combined; H, liver; R, kidneys; T, peripheral tissue; U, urine.

*A*(*t*) of the individual regions of interest were fitted with custom-written MATLAB scripts (MathWorks, Natick, MA) according to the compartment model shown in Figure 2, using the ode45 function to solve the differential equation system. Tracer input was simulated as a constant input of duration *T* (*A*(0)/*T*). Fitting was performed with the solver fmincon and the function MultiStart with 128 random sets of bounded initial parameters. Calculations were performed by parallel computing on 40 cores of the Euler cluster of ETH Zurich (https://scicomp.ethz.ch/). The initial parameters were best guesses from supervised simulations. Initial lower bounds were 0 for the rate constants of mass transfer (*k*). For *k* from plasma to liver and kidneys, the initial upper bounds were set according to the reported values for blood flow [26], corrected for (1 − hematocrit) to get the plasma flow (*Q*_P_) divided by the plasma volume (*V*_Plasma_ = *v*_Blood_  × (1 − hematocrit) × BW). The *Q*_P_ for liver (*Q*_P,H_) was 1.0 ml/min and for kidneys (*Q*_P,R_) 0.73 ml/min.

During the fitting procedure, the sum of weighted squared residuals of all *A*(*t*) was minimized. For weighting, the residuals of the first two data points of plasma, liver, and kidney were multiplied with 5, to force the fits through these initial data points. After several rounds, the best estimates with the lowest sum of squared residuals were used per tracer to define the final upper and lower bounds as 0.5-fold the minimal respective fit parameter and 2-fold the highest respective fit parameter per tracer. This resulted in reproducible fit parameters at a minimal sum of squared residuals for all scans. Under these refined conditions, one calculation (one scan) with 128 random sets of initial values required 10–120 min.

CL_H_ was calculated according to

(2) $$\begin{array}{c} {CL}_{H}=\frac{k_{{BH}_{1}}\times k_{H_{1}H_{2}}}{\left(k_{H_{1}B}+k_{H_{1}H_{2}}\right)}\times V_{Plasma}, \end{array}$$

where the rate constants *k* are defined in the model in Figure 2. Note that at steady state this equals CL_H_ calculated from *k*_H_2_G_.

To calculate CL_R_, the compartments R_1_ and R_2_ were treated as one compartment to reveal (3) in analogy to the previously suggested simplifications [27, 28].

(3) CLR=kBR1kR1B+kR1U/1+kR1R2/kR2R1∗kR1U1+kR1R2/kR2R1×VPlasma.

The term 1/(1 + *k*_R_1_R_2__/*k*_R_2_R_1__) corrects for the mass ratio between R_1_ and the combined R_1_ and R_2_.

Total CL was calculated as the sum of CL_H_ and CL_R_ and compared to the CL estimated from the *C*_Plasma_(*t*) biexponential fits (see (1)), as CL = *λ*_*z*_ × *V*_*z*_. The extraction ratio *E*_H_ for liver was estimated as ratio between CL_H_ and *Q*_P,H_, with *Q*_P,H_ from Davies and Morris [26].

The distribution coefficient between tissue and plasma at equilibrium (*D*_Tissue_) was calculated according to

(4) DTissue=fBT1×kBTkTB1+1−fBT1×kBTkTB2×VPlasmaVTissue.

### 2.4. Statistics

Fit parameters of the individual conditions were compared by homoscedastic 2-tailed Student's *t*-test and differences were defined as significant at *p* < 0.05.

## 3. Results

### 3.1. PET Images with [^11^C]AM7 and [^11^C]MT107

**Figure 3:**
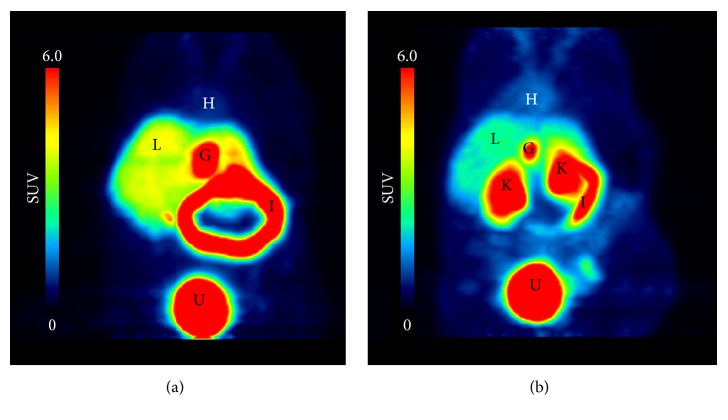
PET images (maximal intensity projections) of mice with (a) 13.9 MBq [^11^C]AM7 and (b) 12.5 MBq [^11^C]AM7 after cyclosporine administration (50 mg/kg i.v.). Radioactivity in the images (*C*(*t*)) was normalized to *A*(0)/BW (standardized uptake value, SUV) and averaged for the complete scan duration of 60 min. G, gallbladder; H, heart; I, intestines; K, kidney; L, liver; U, urinary bladder. CD1 nu/nu mice.

PET images (maximal intensity projections) of [^11^C]AM7 averaged over the complete scan duration are shown in Figure 3. Under control conditions, [^11^C]AM7 radioactivity accumulated in the liver, gallbladder, intestines, and the urinary bladder while the radioactivity in peripheral tissues was negligible (Figure 3(a)). The high radioactivity in gallbladder and intestines is typical for transporter-mediated efflux into bile. The radioactivity distribution changed when cyclosporine, an inhibitor of several human and rodent hepatic transporters, such as P-glycoprotein, OATP1B1, OATP1B3, and BCRP [29], was administered before the tracer. The radioactivity was increased in kidneys and peripheral tissue and reduced in liver, gallbladder, and intestines (Figure 3(b)), indicating a reduction in CL_H_ by cyclosporine.

**Figure 4:**
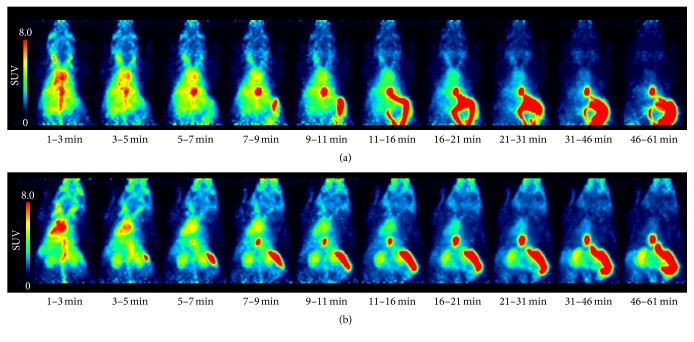
PET images over time of [^11^C]MT107 (SUV, maximal intensity projections). (a) Control scan, 7.1 MBq [^11^C]MT107. (b) Scan after cyclosporine treatment (50 mg/kg, i.v.), 11.7 MBq [^11^C]MT107. The time windows are indicated. SCID mice.

The findings were similar for the [^11^C]AM7 derivative [^11^C]MT107.  Figure 4 shows PET images (maximal intensity projections) of [^11^C]MT107 over time. Cyclosporine administration before the injection of [^11^C]MT107 resulted in an increased radioactivity uptake in the kidneys and peripheral tissue and reduction in the liver as compared to scans without cyclosporine. The respective images averaged over the complete scan duration are shown in Supplementary Figure 2.

### 3.2. Kinetics of the Tracers in Blood Plasma

**Figure 5:**
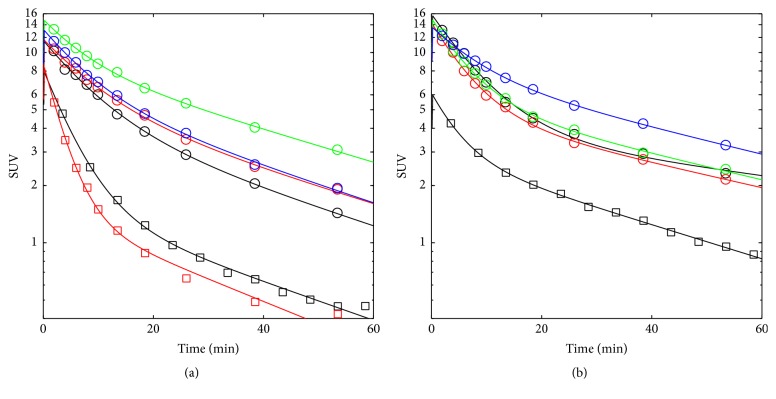
SUV plasma curves of [^11^C]AM7 and [^11^C]MT107 as derived from the PET images. (a) Control (circles, [^11C^]MT107, *n* = 4; squares, [^11^C]AM7, *n* = 2). (b) After cyclosporine treatment (*n* = 4 for [^11C^]MT107; *n* = 1 for [^11^C]AM7). Lines, biexponential fits according to (1). Colours distinguish individual data sets. Fit parameters; see Table 1.

**Figure 6:**
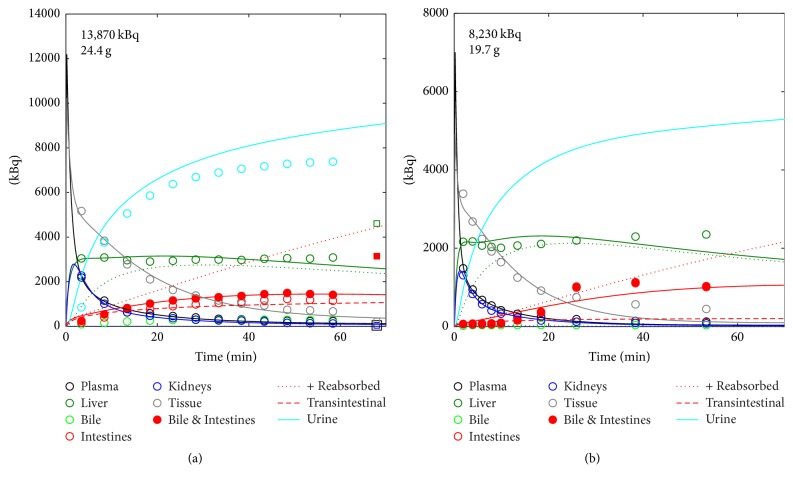
Experimental and fit *A*(*t*) of [^11^C]AM7 under control conditions. (a) Scan with available image data for urine and with blood and tissue data from dissection. (b) Scan without control data from dissection and no complete urinary bladder in the images. Circles, image data; squares, data from dissection ((a), at 68 min). Lines, fits of the image data. Colour code, see insert. Dotted green line, simulated fraction of *A*_Liver_(*t*) in compartment H_2_ (see Figure 2). Dotted red line (+Reabsorbed), sum of the compartments G and the fraction reabsorbed according to *k*_GH_1__ (Figure 2). Broken red line (Transintestinal), simulated tracer excretion from plasma to intestines, according to *k*_BG_ in Figure 2. Note that urinary data in (a) (light blue circles) were not fitted but were predicted from the modelling (light blue line). *A*(0) and BW are indicated in the panels. CD1 nu/nu mice.

**Figure 7:**
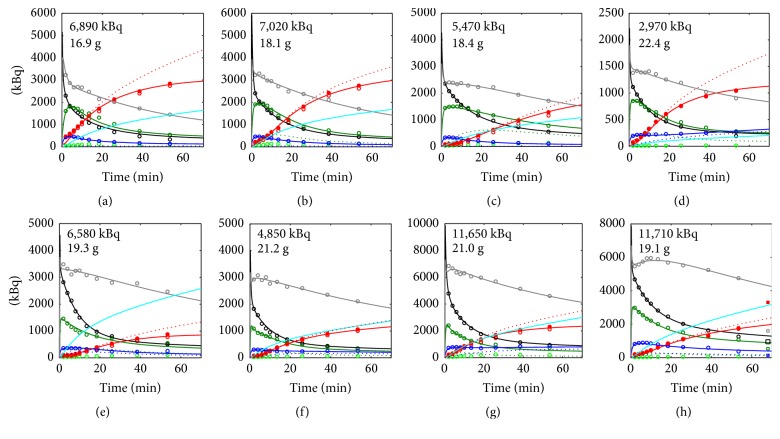
[^11^C]MT107 experimental and modelled *A*(*t*) of blood plasma, liver, gallbladder, intestines, kidneys, peripheral tissue, and urine (modelled only). (a–d) [^11^C]MT107 under control conditions. Vehicle (13% ethanol, 2 ml/kg) was administered 30 min before tracer to the animal in (d) as a control. (e–h) [^11^C]MT107 after cyclosporine treatment. Circles, experimental data derived from the PET images. Squares (h), available experimental data from dissection at the end of the scan (68 min). Lines, modelled *A*(*t*). Colours and line style, see Figure 6. *A*(0) and BW are indicated in the panels. The respective *C*(*t*) curves are shown in Supplementary Figure 4. SCID mice.

Figure 5 shows *C*_Plasma_(*t*) as derived from the PET images with the respective biexponential fits (see (1)) for [^11^C]AM7 and [^11^C]MT107 under baseline conditions and after the administration of cyclosporine. The fit parameters are shown in Table 1. Note that *C*_Plasma_(*t*) may be underestimated and *V*_1_ and *V*_*z*_ accordingly overestimated, due to radioactivity spill-over and partial volume effects (see Section 2.2). However, we did not find a major disagreement between the blood radioactivity of the last image time window and as determined from the dissection experiments (Figures 6(a), 7(h) and Supplementary Figure 3).

For both tracers, *λ*_*z*_ was reduced in the presence of cyclosporine resulting in a prolonged half-life (*t*_1/2_ = ln⁡(2)/*λ*_*z*_), significant for [^11^C]MT107 (Table 1). The CL was reduced by trend, but not at the significance level (*p* = 0.23). For [^11^C]MT107, it was 56.1 ± 1.3 *μ*l/min under control and 45.0  ±  1.0 *μ*l/min under cyclosporine conditions. For comparison, the maximal expected CL by glomerular filtration would be ~160 *μ*l/min, the maximal possible CL_H_ ~1000 *μ*l/min, and the maximal CL by renal filtration with additional transporter-mediated renal excretion ~730 *μ*l/min, according to the reported respective values for *Q*_P_ [26].

### 3.3. Physiologically Based Pharmacokinetic Modelling

We first evaluated the modelling according to the model in Figure 2 with data from a control [^11^C]AM7 scan which included the complete urinary bladder and for which data from dissection were available (scan in Figure 3(a)). We found a good agreement between the fitted and the experimental data (Figure 6). *A*_urine_(*t*) as derived from the PET images was not used for the fitting, as it was not available in the remaining data sets. The good agreement between the predicted *A*_urine_(*t*) from the modelling and the experimental data further confirmed the accuracy of the modelling. In addition, results from the two [^11^C]AM7 control scans were consistent (Figure 6, Table 1). Compared with the results from the dissection, radioactivities of liver and combined gallbladder and intestines were underestimated from the PET images while tissue radioactivity was higher from the PET images (shoulder, Supplementary Figure 1) than the dissection (vastus lateralis and rectus femoris). The fit parameters are shown in Table 1 and Supplementary Table 1. The data of the [^11^C]AM7 scan after cyclosporine administration shown in Figure 3(b) were not suitable for modelling as *C*_kidney_(*t*) was poorly defined (Supplementary Figure 3 and Supplementary Table 1).

Figure 7 shows the image-derived *A*(*t*) and computed fits for [^11^C]MT107 under control conditions and after cyclosporine treatment. The respective *C*(*t*) are shown in Supplementary Figure 4. For one scan (Figure 7(h)), data from dissection were available indicating an underestimation of intestinal radioactivity in the images. As concluded from Figure 7, the accumulation in liver was reduced after cyclosporine treatment. Under control conditions, the image-derived *A*(*t*) for the combined gallbladder and intestines exceeded the modelled *A*_urine_(*t*). This was not the case after cyclosporine treatment. This indicates that the radioactivity was mainly cleared by hepatobiliary excretion under control conditions but not after cyclosporine treatment.

The fit parameters of [^11^C]AM7 and [^11^C]MT107 are shown in Table 1 and Supplementary Table 1. Under control conditions, CL_H_ was in the range of 100 *μ*l/min for [^11^C]AM7 and 35.2 ± 10.9 *μ*l/min for [^11^C]MT107. This is low compared to *Q*_P,H_, the maximal possible CL_H_ (~1000 *μ*l/min). As a consequence, the values of *E*_H_ were low for both tracers. After cyclosporine treatment, CL_H_ and *E*_H_ of [^11^C]MT107 were significantly reduced to 48% of the respective values in the absence of cyclosporine (Table 1). For *E*_H_, this corresponded to an averaged reduction from 0.035 ± 0.011 to 0.017 ± 0.006 (*p* = 0.025).

The CL_R_ calculated for the two [^11^C]AM7 control scans was in the range of the reported glomerular filtration rate (GFR) of 160 *μ*l/min [26]. In the case of [^11^C]MT107, it was lower than the GFR with CL_R_/GFR fractions <0.2 for all scans. CL_R_/GFR < 1 could result from a reduced filtration due to plasma protein binding or from reabsorption of the tracer after glomerular filtration. A difference in renal reabsorption between [^11^C]AM7 and [^11^C]MT107 would be expected from their difference in lipophilicity, that is, log⁡*D* (pH 7.4) 0.1 versus 2.0 [30].

After cyclosporine administration, CL_R_ of [^11^C]MT107 was increased 1.7-fold on average, though not at the significance level (*p* = 0.069). As a consequence of the significant reduction in CL_H_ and tentative increase in CL_R_ after cyclosporine treatment, the averaged ratio CL_H_/CL_R_ decreased from 3.8 ± 3.0 to 0.9 ± 0.2 (*p* = 0.11). The excretion pattern changed from preferentially hepatobiliary to similar contributions from both hepatobiliary and renal pathways.

For both tracers, the fits were best when including an irreversible besides a reversible uptake into liver (Figure 2) with radioactivity excretion from the irreversible compartment to gallbladder and intestine. The fits and results were similar if the two compartments were in parallel, both adjacent to the plasma compartment (data not shown). For the kidneys, two reversible compartments revealed best fits with excretion into urine from the compartment adjacent to plasma. As observed for liver, the fits and results were similar when the kidney compartments were arranged both adjacent to the plasma compartment (data not shown). The irreversible uptake into the liver before excretion is in agreement with transporter-mediated irreversible uptake into hepatocytes or efflux into the canaliculi. For kidney, the two reversible compartments could reflect reversible distribution into the kidneys and glomerular filtration for compartment R_1_ and tracer accumulation by the concentrating primary urine and reabsorption from the tubuli for compartment R_2_.

Besides the tentative increase in CL_R_, the average *D*_Tissue_ of [^11^C]MT107 was nonsignificantly increased by a factor of 1.34 after cyclosporine administration (*p* = 0.078), in agreement with the trend of *V*_*z*_, which increased 1.25-fold (*p* = 0.21). Besides transporter inhibition, cyclosporine can displace drugs from plasma protein binding [31, 32]. An increase in *f*_u_ of [^11^C]MT107 would explain both the increase in *D*_Tissue_ and in CL_R_. We did not further investigate this since the effects on CL_R_ and *D*_Tissue_ were not significant. The relatively high CL_R_ of [^11^C]AM7 in the range of the GFR would indicate that *f*_u_ is not limiting for glomerular filtration, at least for [^11^C]AM7.

In our model, *k*_GH_1__ is the rate constant of reabsorption from intestines by portal vein into the reversible compartment of the liver (H_1_ in Figure 2). We hypothesized that inhibition of efflux transporters in the intestinal mucosa by cyclosporine may increase reabsorption and, therefore, *k*_GH_1__. However, the averaged *k*_GH_1__ for [^11^C]MT107 in the absence and presence of cyclosporine did not differ, they were 0.0077 ± 0.0033 min^−1^ and 0.0080 ± 0.0038 min^−1^, respectively (*p* = 0.90). The simulated reabsorbed fractions of [^11^C]MT107 are indicated in Figure 7. It should, however, be noted that *k*_GH_1__ could alternatively or in addition compensate for* A*(*t*) under- or overestimations, in particular as image-derived *A*(*t*) and *A*(*t*) from dissection were not in full agreement.

In the above calculations, *k*_BG_, defining the transintestinal excretion from plasma to intestines, was set to 0 (Figure 2). When *k*_BG_ was fitted for [^11^C]MT107 scans, average CL_H_ (ml/min) were 25.4  ±  6.0 under control conditions and 9.3 ± 4.2 after cyclosporine administration with a significant difference (*p* = 0.0045). The calculated transintestinal clearance (ml/min) varied between the scans with 10.5 ± 11.3 for the control and 7.8 ± 4.1 for the cyclosporine group (*p* = 0.67). The sum of the individual CL_H_ and transintestinal clearance was similar to the CL_H_ calculated with *k*_BG_ = 0, including *k*_BG_ in the model improved the fit of liver *A*(*t*) in Figure 7(a).

The modelling revealed a 16% (control group) and 17% (cyclosporine group) lower total CL of [^11^C]MT107 than the biexponential fit of *C*_Plasma_(*t*) (Table 1). The differences between the two methods were not significant (*p* > 0.27). They could result from an overestimation of *V*_*z*_ as discussed in Section 2.2 and from errors in estimating *A*(*t*) for the individual organs and tissues from the image data in general, as several assumptions were made on organ and tissue volumes and as PET data are biased by partial volume effects and radioactivity spill-over [33].

## 4. Discussion

We demonstrated that PBPK modelling is feasible with dynamic mouse PET data. In our case, we apply this analysis to guide the further development of [^11^C]AM7-derived PET tracers for the purpose of imaging hCD80 levels by PET. We suggest that this method can further be used to study the influence of drugs on transporter activity and on the pharmacokinetics in general, by applying PET tracers which are substrates of the saturable pharmacokinetic process of interest. Besides these applications, PBPK modelling in combination with nuclear imaging was successfully demonstrated for ^177^Lu-DOTATATE used for therapy in patients with neuroendocrine tumours [34]. The authors suggested to apply PBPK to model the biodistribution and absorbed radiation doses of therapeutic radiotracers in the healthy and tumour tissues of patients in order to better evaluate the risk/benefit balance and find the optimal radioactivity dose for tumour treatment.

Several protocols exist to evaluate hepatobiliary excretion, and transporter activity by PET or SPECT [11, 35]. These methods use simplified models, possibly revealing more robust results and requiring less computing capacity than the full-compartment modelling presented here. In contrast to the simplified models which focus on one particular organ and its function, full compartment modelling allows identifying distinct alterations in a more complex model where all relevant processes can be included, for example, tissue distribution and renal excretion in addition to hepatobiliary excretion, as in the presented example. To assure the applicability of our model, we kept the number of compartments and rate constants to a minimum, while keeping focus on reliable fitting results.

For this study, we repurposed data from previous PET experiments. The experiments were not originally designed for PBPK modelling. For this reason, we encountered some limitations which have to be taken into consideration when planning a PBPK study by PET. (i) Based on the design of our control experiments, we cannot exclude that the adjuvants in Sandimmun had an influence on the pharmacokinetics of our tracers. (ii) The first minute after tracer injection is not included in our experimental data. This time window is essential for the accurate modelling of rate constants between plasma and tissues. (iii) Having the urinary bladder in the field of view would allow estimating the amount of radioactivity in urine and comparing it with the modelling results or include it in the fitting. Depending on the size of the field of view, this is not always possible. In our study, we used the available data of urinary bladder of one scan to evaluate the modelling and found a good agreement between the predicted and experimental data. (iv) Owing to the small *V*_1_ and *V*_*z*_ of the two tracers, we were able to estimate *C*_Blood_(*t*) from the image data. Ideally, *C*_Blood_(*t*) is determined from an arteriovenous shunt to avoid bias by partial volume effects and radioactivity spill-over and to get a high temporal resolution [13, 36]. (v) A further limitation of this study is the low number of animals scanned with [^11^C]AM7 where two scans were available for control conditions and no reliable data was available to study the influence of cyclosporine by PBPK modelling. Therefore, no statistical analysis could be applied to compare the two tracers.

Tracer metabolism should be negligible when studying transporter activity. In particular, as cyclosporine is not only an inhibitor of drug transporters but also of the drug-metabolising enzyme CYP3A4 in humans [29]. Radiometabolite formation would complicate the modelling. No radiometabolites were detected in the blood plasma 30 min after [^11^C]AM7 administration in our previous study [16]. Demethylation of the [^11^C]methyl group of both tracers by cytochrome P450 would be the most probable radiometabolite-forming reaction [37, 38]. The resulting radiometabolite [^11^C]formaldehyde or its oxidation and reduction products would accumulate in bone marrow and salivary gland besides liver [39]. We did not find such radioactivity distribution in our PET images, excluding major [^11^C]demethylation of the tracers.

As pointed out by Stieger et al. [35], studies as presented here can contribute to a better understanding of the mechanisms of drug-drug interactions and can provide information for the generation of model parameters for PBPK modelling based on* in vitro* data. In the future, rodent and human PET with dedicated tracers will support the building and refinement of PBPK models to facilitate the translation from* in vitro* to the* in vivo* preclinical phase and to support the prediction of the pharmacokinetics in humans based on preclinical and clinical data. Besides a calibrated PET scanner with high spatial resolution, high computing capacity and parallel computing are favourable for successful modelling.

## 5. Conclusions

By PBPK modelling using dynamic PET data from mice, we were able to characterize distinct pharmacokinetic details for two structurally related radiotracers. Our modelling approach allowed identification of the pharmacokinetic alterations induced by the transporter inhibitor cyclosporine. Our study shows the potential of PBPK modelling with PET data for radiotracer and drug development, as well as for evaluating and predicting the effects of transporter inhibition on whole-body pharmacokinetics.

## Figures and Tables

**Table 1:** Plasma pharmacokinetics and PBPK modelling results based on [^11^C]AM7 and [^11^C]MT107 PET data.

	[^11^C]AM7		[^11^C]MT107	
	Control (*n* = 2)^a^	Cyclosporine (*n* = 1)^a^	Control (*n* = 4)^a^	Cyclosporine (*n* = 4)^a^
Body weight (g)	24.4/19.7	25.3	19.0 ± 2.4	20.2 ± 1.1
*Plasma kinetics (Equation (1))*				
*V*_1_ (ml)	3.0/2.3	4.2	1.49 ± 0.19	1.38 ± 0.11
*V*_*z*_ (ml)	10.3/9.4	8.0	2.66 ± 0.51	3.33 ± 0.80
*λ*_1_ (1/min)	0.196/0.295	0.204	0.121 ± 0.011	0.139 ± 0.021
*λ*_*z*_ (1/min)	0.0234/0.0270	0.0206	0.0210 ± 0.0011	0.0140 ± 0.0034^*∗∗*b^
CL (*µ*l/min)	242/253	165	56.1 ± 13.0	45.0 ± 10.1
*In vivo PBPK modelling*				
CL_H_ (*µ*l/min)	103 (18.3%)^c^/120 (5.8%)^c^	n.d.^d^	35.2 ± 10.9	17.1 ± 5.6^*∗*b^
*E*_H_ (-)		n.d.	0.035 ± 0.011	0.017 ± 0.006^*∗*b^
CL_R_ (*µ*l/min)	161/179	n.d.	11.9 ± 5.3	19.9 ± 5.1
CL_R_/GFR (-)	1.0/1.1	n.d.	0.074 ± 0.033	0.12 ± 0.03
CL_H_/CL_R_ (-)	0.64/0.63	n.d.	3.8 ± 3.0^e^	0.9 ± 0.2^f^
CL (*µ*l/min)	264/298	n.d.	47.1 ± 11.9	37.0 ± 8.9
*D*_Tissue_ (-)	0.11/0.12	n.d.	0.10 ± 0.02	0.14 ± 0.03

PBPK parameters were calculated from the fitted *k* according to (2) and (3) and the model in Figure 2. ^a^Individual values for [^11^C]AM7 and mean with standard deviations for [^11^C]MT107; *n*, number of scans; ^b^significant decrease compared to [^11^C]MT107 control; ^c^for [^11^C]AM7, CL_H_ includes *k*_BG_ × *V*_Plasma_ (% contribution shown in brackets; see Figure 2) ^d^n.d., not determined; ^e^all ratios > 2.0; ^f^all ratios < 1.1. ^*∗*^*p* < 0.05; ^*∗∗*^*p* < 0.01.

## Data Availability

Raw data and metadata of the PET scans as well as the MATLAB scripts are available from the corresponding author on request. Data are still under evaluation by the authors for other purposes (Taddio et al., in preparation).

## Supplementary Materials

Supplementary Materials Supplementary PDF file includes the following: (i) Supplementary Figure 1: volumes of interest for the kinetic modelling. (ii) Supplementary Figure 2: PET images (maximal intensity projections) of mice with [^11^C]MT107 and [^11^C]MT107 after cyclosporine administration (50 mg/kg i.v.). The same data as in Figure 4 but averaged for the complete scan duration. (iii) Supplementary Figure 3: *A*(*t*) experimental data and fits of the [^11^C]AM7 scan after cyclosporine treatment in Figure 3(b). (iv) Supplementary Figure 4: *C*(*t*) of the scans and fits in Figure 7. (v) Supplementary Table 1: fit parameters.
