# Supplementary material for: Physiologically Based Pharmacokinetic Modelling with Dynamic PET Data to Study the In Vivo Effects of Transporter Inhibition on Hepatobiliary Clearance in Mice
Source: Contrast Media Mol Imaging. 2018 Jun 3;2018:5849047. doi: 10.1155/2018/5849047 (PMC6008768; doi:10.1155/2018/5849047)
Supplement: Supplementary Materials — Supplementary PDF file includes the following: (i) Supplementary Figure 1: volumes of interest for the kinetic modelling. (ii) Supplementary Figure 2: PET images (maximal intensity projections) of mice with [11C]MT107 and [11C]MT107 after cyclosporine administration (50 mg/kg i.v.). The same data as in Figure 4 but averaged for the complete scan duration. (iii) Supplementary Figure 3: A(t) experimental data and fits of the [11C]AM7 scan after cyclosporine treatment in Figure 3(b). (iv) Supplementary Figure 4: C(t) of the scans and fits in Figure 7. (v) Supplementary Table 1: fit parameters. [file 5849047.f1.pdf]

Physiologically-based pharmacokinetic modelling (PBPK) with dynamic PET data to study the *in vivo* effects of transporter inhibition on hepatobiliary clearance in mice

Supplementary Figures 1 – 4
Supplementary Table 1

Marco F. Taddio [1], Linjing Mu [2], Claudia Keller [1], Roger Schibli [1], Stefanie D. Krämer [1]

[1] Radiopharmaceutical Science and Biopharmacy, Institute of Pharmaceutical Sciences, Department of Chemistry and Applied Biosciences, ETH Zurich, Zurich, Switzerland

[2] Department of Nuclear Medicine, University Hospital Zurich, Switzerland

Corresponding author:

Stefanie Krämer
Radiopharmaceutical Science and Biopharmacy
Institute of Pharmaceutical Sciences
Department of Chemistry and Applied Biosciences
ETH Zurich
Vladimir-Prelog-Weg 4
CH-8093 Zurich
Switzerland
skraemer@pharma.ethz.ch

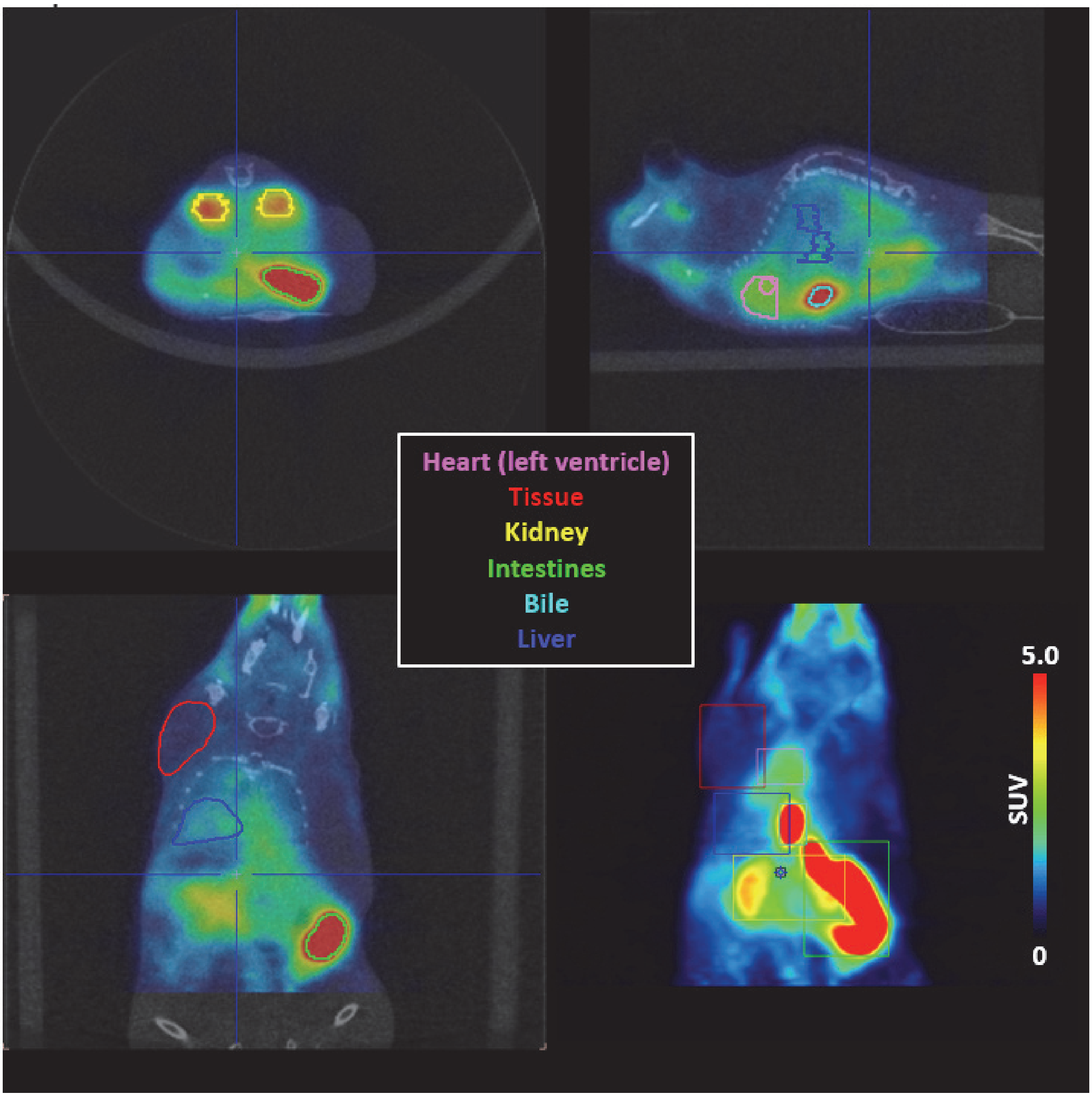

Supplementary Figure 1. Volumes of interest for the kinetic modelling. The heart left ventricle volume was generated with the PSEG module of the software PMOD as described in the section “Time activity curves”. All other volumes of interest were generated with the VOI tools of PMOD.

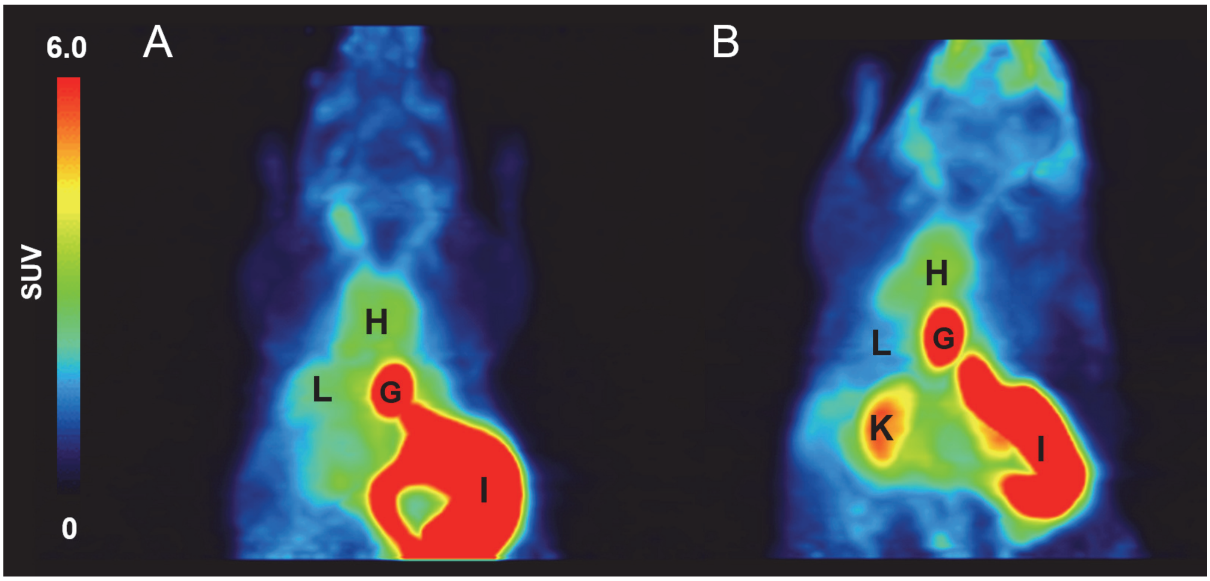

Supplementary Figure 2. PET images (maximal intensity projections) of mice with **A**) [^{11}C]MT107, **B**) [^{11}C]MT107 after cyclosporine administration (50 mg/kg i.v.). Same data as in **Figure 4** but averaged for the complete scan duration. G, gallbladder; H, heart; I, intestines; K, kidney; L, liver; U, urinary bladder.

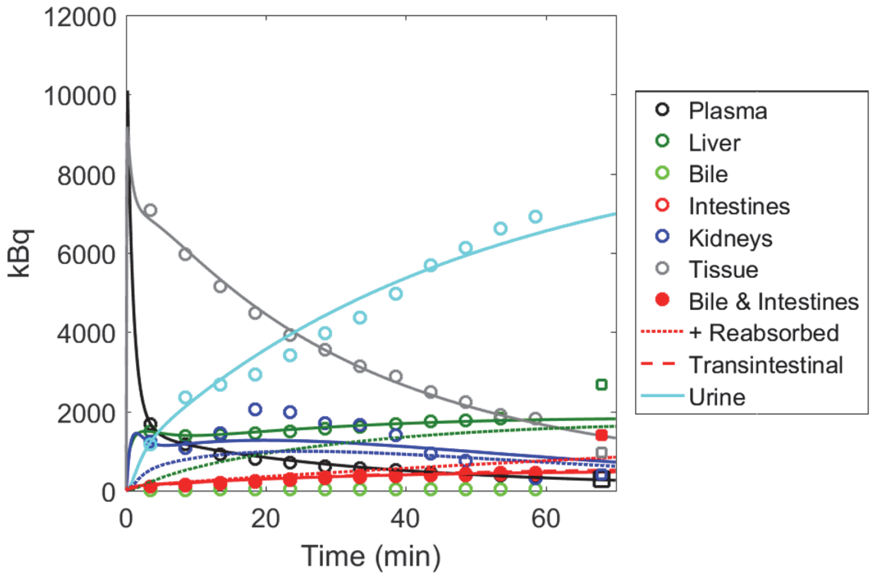

Supplementary Figure 3. *A*(*t*) experimental data and fits of the [^{11}C]AM7 scan after cyclosporine treatment in **Figure 3B**. Note that the experimental $A_{kidney}(t)$ was not well defined resulting in unreliable fitting results. The fitted parameters are shown in **Supplementary Table 1**. CD1 nu/nu mouse.

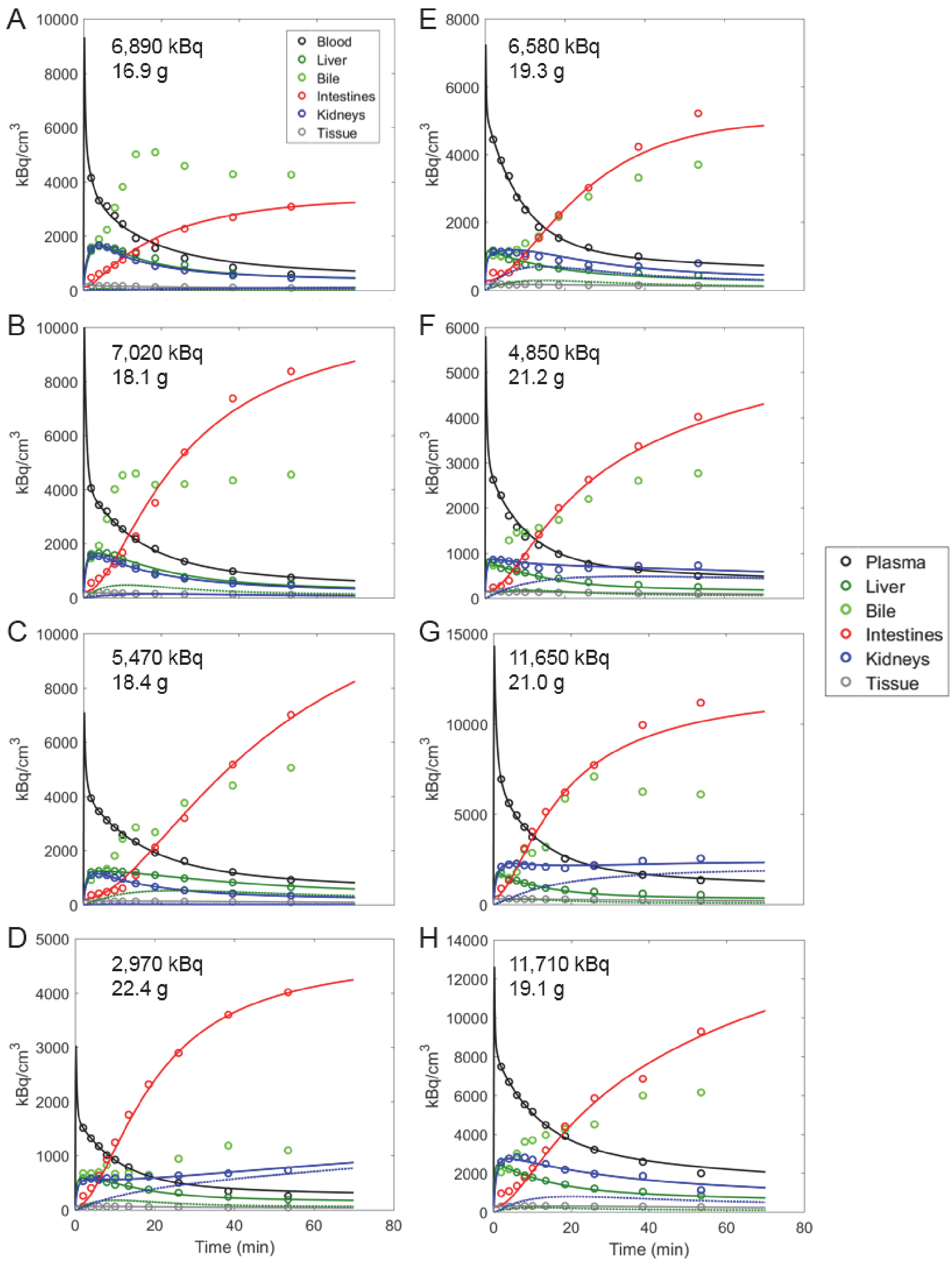

Supplementary Figure 4. $C(t)$ of the scans and fits in Figure 7. Note that the fits were optimized for minimal squared residues in $A(t)$.

Supplementary Table 1. Fit parameters

																					Parameters from bi-exponential fit of CPlasma(t)					Calculated parameters from modelling							
	BW	kBH1	KH1B	kH1H2	kBR1	kR1B	kR1R2	kBT	kT1B	1-fBT1	kT2B	kH2G	kBG	kGH1	kR2R1	kR1U	vPlasma	T	VTissue/BW	Sum normalized squared residues	V1	Vz	l1	lz	CL from CPlasma(t)	CLR	CLH	CL BG	CL total	CLH/CLR	Dtissue	E	CLR/GFR
AM7 Control																																	
Fig. 6A	24.4	0.2595	0.2306	0.1569	0.3819	0.2497	0	0.8149	163.382	0.3038	0.1222	0.0193	0.0236	0.0397	0.2676	0.2796	0.033	0.15	0.6001	1.1925	3.0316	10.3465	0.1963	0.0234	241.854	161.258	83.97	18.826	264.054	0.637	0.114	0.084	1.008
Fig. 6B	19.7	0.5099	0.3992	0.208	0.4691	0.2939	0.0035	0.4235	110.3334	1	0.1948	0.0145	0.0107	0.0226	0.0655	0.4241	0.033	0.15	0.6	3.572	2.2853	9.3597	0.2955	0.0271	253.191	178.824	112.742	6.888	298.454	0.63	0.119	0.113	1.118
AM7 Cyclosporin																																	
Suppl.Fig. 3	25.3	0.1757	0.2414	0.062	0.2939	0.4502	0.1798	0.5934	146.4993	1	0.1516	0.0037	0.0093	0.0161	0.0459	0.3665	0.033	0.15	0.745	3.7216	4.1679	8.0049	0.2044	0.0206	164.996	109.306	29.737	7.69	146.732	0.272	0.172	0.03	0.683
MT107 control		k1H	k2H	k3H	k1R	k2R	k3R					k5H			k4	k5																	
Fig. 7A	16.9	0.3825	0.3087	0.0716	0.1178	0.3626	0.0023	1.7587	2.3107	0.0344	0.0425	1.9446	0	0.0096	0.0002	0.1172	0.033	0.15	0.7781	0.8004	1.4672	2.9144	0.1156	0.0217	63.178	15.935	39.853	0	55.788	2.501	0.091	0.04	0.1
Fig. 7B	18.1	0.5365	0.666	0.0843	0.1185	0.4322	0.012	0.5458	0.7509	0.1141	0.0327	0.1573	0	0.0047	0.0642	0.1318	0.033	0.15	0.6198	0.3567	1.546	2.7537	0.1102	0.0204	56.041	16.427	35.74	0	52.167	2.176	0.135	0.036	0.103
Fig. 7C	18.4	0.5769	0.942	0.0572	0.1108	0.5968	0.0045	1.1588	3.6017	0.0474	0.0312	0.0509	0	0.0052	0.1438	0.1009	0.033	0.15	0.769	0.097	1.2381	1.9231	0.1345	0.0198	37.989	9.664	19.915	0	29.579	2.061	0.088	0.02	0.06
Fig. 7D	22.4	1.0984	1.6095	0.0959	0.1654	0.9025	0.0633	1.0125	2.3428	0.0797	0.037	0.1601	0	0.0113	0.0003	0.0424	0.033	0.15	0.8033	0.1801	1.6977	3.0572	0.1244	0.022	67.37	5.445	45.348	0	50.793	8.329	0.105	0.045	0.034
Average	18.95	0.6486	0.8815	0.0773	0.1281	0.5735	0.0205	1.119	2.2515	0.0689	0.0359	0.5782	0	0.0077	0.0521	0.0981	0.033	0.15	0.7425	0.3586	1.4872	2.6621	0.1211	0.021	56.145	11.868	35.214	0	47.082	3.767	0.105	0.035	0.074
Stdev	2.39	0.3114	0.5502	0.0167	0.0251	0.2403	0.0288	0.5002	1.1671	0.0357	0.005	0.9123	0	0.0033	0.0682	0.0392	0	0	0.0831	0.3138	0.1917	0.508	0.0107	0.0011	12.976	5.274	10.932	0	11.857	3.047	0.021	0.011	0.033
MT107 Ciclosporin																																	
Fig. 7E	19.3	1.4079	3.1816	0.0529	0.2	1.6135	0.1559	1.8242	4.78	0.0499	0.03	0.0736	0	0.0126	0.0806	0.4096	0.033	0.15	0.693	0.6393	1.2217	4.105	0.1222	0.0089	36.526	25.599	14.546	0	40.146	0.568	0.161	0.015	0.16
Fig. 7F	21.2	1.1645	2.396	0.0688	0.161	0.8661	0.0634	0.7726	0.9772	0.153	0.0335	0.1381	0	0.0048	0.0243	0.208	0.033	0.15	0.9224	0.5452	1.4899	3.7155	0.1589	0.0151	56.088	21.657	22.589	0	44.246	1.043	0.149	0.023	0.135
Fig. 7G	21	0.6413	1.6058	0.079	0.0874	0.3313	0.054	0.3913	0.5014	0.2265	0.031	0.2424	0	0.01	0.0107	0.1526	0.033	0.15	0.7706	0.8965	1.3982	3.2283	0.1558	0.0159	51.185	18.963	20.681	0	39.643	1.091	0.147	0.021	0.119
Fig. 7H	19.1	1.4933	2.5142	0.0286	0.1821	0.886	0.0471	2.1846	5.0072	0.0369	0.0397	0.1761	0	0.0049	0.0759	0.1194	0.033	0.15	0.7816	0.3211	1.4007	2.2637	0.121	0.0161	36.363	13.536	10.498	0	24.034	0.776	0.103	0.01	0.085
Average	20.15	1.1767	2.4244	0.0573	0.1576	0.9242	0.0801	1.2932	2.8165	0.1166	0.0335	0.1576	0	0.008	0.0479	0.2224	0.033	0.15	0.7919	0.6005	1.3776	3.3281	0.1395	0.014	45.04	19.939	17.078	0	37.017	0.869	0.14	0.017	0.125
Stdev	1.1	0.3832	0.6461	0.022	0.0495	0.5265	0.051	0.8487	2.4081	0.0898	0.0043	0.0706	0	0.0039	0.0356	0.13	0	0	0.0955	0.2382	0.1124	0.7951	0.0207	0.0034	10.126	5.064	5.569	0	8.898	0.244	0.025	0.006	0.032
ttest	0.397	0.0762	0.0109	0.1982	0.3284	0.2711	0.0881	0.7356	0.6876	0.3621	0.511	0.3933		0.898	0.9158	0.1168			0.4653	0.2653	0.3618	0.2077	0.166	0.008	0.2259	0.0694	0.0254		0.2234	0.1068	0.0779	0.0254	0.0694

vPlasma	0.03276	ml/g
QH	1000	µl/min
QR	730	µl/min
GFR	160	µl/min
